# Healthcare-associated cluster of *Burkholderia sola* in two lung transplant recipients characterized through whole-genome sequencing

**DOI:** 10.1017/ice.2025.10261

**Published:** 2025-10

**Authors:** Alexander Sundermann, Marissa Griffith, Deena Ereifej, Nathan J. Raabe, Vatsala Rangachar Srinivasa, Kady Waggle, Kathleen Shutt, Hunter Coyle, Ashley Ayres, Spencer Schrank, Graham M. Snyder, Daria Van Tyne, Lora Lee Pless, Lee H. Harrison

**Affiliations:** 1 Microbial Genomic Epidemiology Laboratory, Center for Genomic Epidemiology, University of Pittsburgh, Pittsburgh, PA, USA; 2 Department of Epidemiology, School of Public Health, University of Pittsburgh, Pittsburgh, PA, USA; 3 Division of Infectious Diseases, University of Pittsburgh School of Medicine, Pittsburgh, PA, USA; 4 Department of Infection Control and Hospital Epidemiology, UPMC Presbyterian, Pittsburgh, PA, USA

## Introduction

In early 2025, an astute clinician noted a higher-than-expected frequency of *Burkholderia cepacia* complex infections in lung transplant recipients. Two of these isolates were subsequently characterized by Enhanced Detection System for Healthcare-Associated Transmission (EDS-HAT), our real-time bacterial genomic surveillance system, as genetically indistinguishable *Burkholderia sola* infections.^1^ *B. sola* is a newly described environmental species within the *B. cepacia* complex, with limited documentation of human infection.^2^ Here, we describe the investigation of this healthcare-associated cluster.

## Cluster cases

Patient 1 is a 70-year-old individual with chronic respiratory failure due to idiopathic pulmonary fibrosis, admitted for lung transplant evaluation. A single lung transplantation was performed 36 days after admission. Sterility cultures of the harvested lung immediately prior to transplant grew an isolate identified by the clinical microbiology laboratory as *B. cepacia* complex, using matrix-assisted laser desorption ionization-time of flight spectrometry, as well as *Acinetobacter calcoaceticus* and methicillin-susceptible *Staphylococcus aureus*. On post-transplant day 2, bronchoalveolar lavage (BAL) cultures from the transplanted lung were positive for *B. cepacia* complex. The patient received a 2-week course of antibiotic treatment. Bacteremia with *B. cepacia* complex was detected on post-transplant day 34; the patient was transferred to the intensive care unit (ICU) two days later and subsequently died on post-transplant day 51 from multiorgan failure in the setting of septic shock. Whole genome sequencing was performed on *Burkholderia* isolates from the donor lung sterility culture and bloodstream isolates.

Patient 2 is a 70-year-old individual with interstitial lung disease who underwent single lung transplantation 15 days following hospital admission. Sterility cultures of the harvested lung immediately prior to transplant grew methicillin-susceptible *Staphylococcus aureus* and oropharyngeal flora including yeast, and *Ureaplasma urealyticum* by polymerase chain reaction. Post-operatively, the patient was clinically diagnosed with pneumonia for which the patient received multiple antimicrobials from post-transplant day 6 through 25. BAL cultures on post-transplant days 1, 4, 7, and 23 demonstrated no growth. The patient was transferred on post-transplant day 28 to a long-term acute care facility located within the acute care hospital. The patient continued to have respiratory failure requiring ventilation and underwent a BAL on post-transplant day 43 that grew *B. cepacia* complex for which antibiotics were administered and the patient continues to receive care at the time of this report.

One additional patient with *B. cepacia* complex was identified as spatiotemporally related within the cluster. Patient 3 was transferred from an acute care hospital with heart failure and multiple organ dysfunction, admitted to the same ICU room as Patient 2 (38 days after Patient 2 was transferred from the room), and a BAL culture grew *B. cepacia* complex on hospital day 3.

## Epidemiologic and genomic investigation

All three patients were cared for in the same ICU, Patients 1 and 2 concomitantly. The lung transplant surgeries for Patients 1 and 2 occurred in separate operating rooms with different surgical teams. The bronchoscopy procedures for all three patients entailed no common bronchoscopes or procedural staff. Aside from ICU care, no significant epidemiological commonalities were found between Patient 3 when compared to Patients 1 and 2. An investigation into the organ procurement process revealed no evidence that the lungs for Patient 1 were contaminated during procurement and transport. On retrospective microbiological surveillance, no other patients with laboratory-confirmed *Burkholderia cepacia* complex identified as potentially related to this cluster within several months prior to the index case; at the time of this report, no subsequent cases have been identified. In response to the cluster, supervised terminal cleaning was conducted in both the shared ICU room and operating rooms where Patients 1 and 2 underwent transplantation to ensure thorough environmental decontamination.

Genomic comparison of isolates found 0 SNP differences between isolates from Patients 1 and 2, identified as *B. sola* species (sequence data available at BioProject PRJNA475751). These were genetically unrelated to the isolate from Patient 3, *B. cenocepacia*, and all other sequenced *Burkholderia* isolates at our institution (>36,000 SNPs).

**Figure 1.**
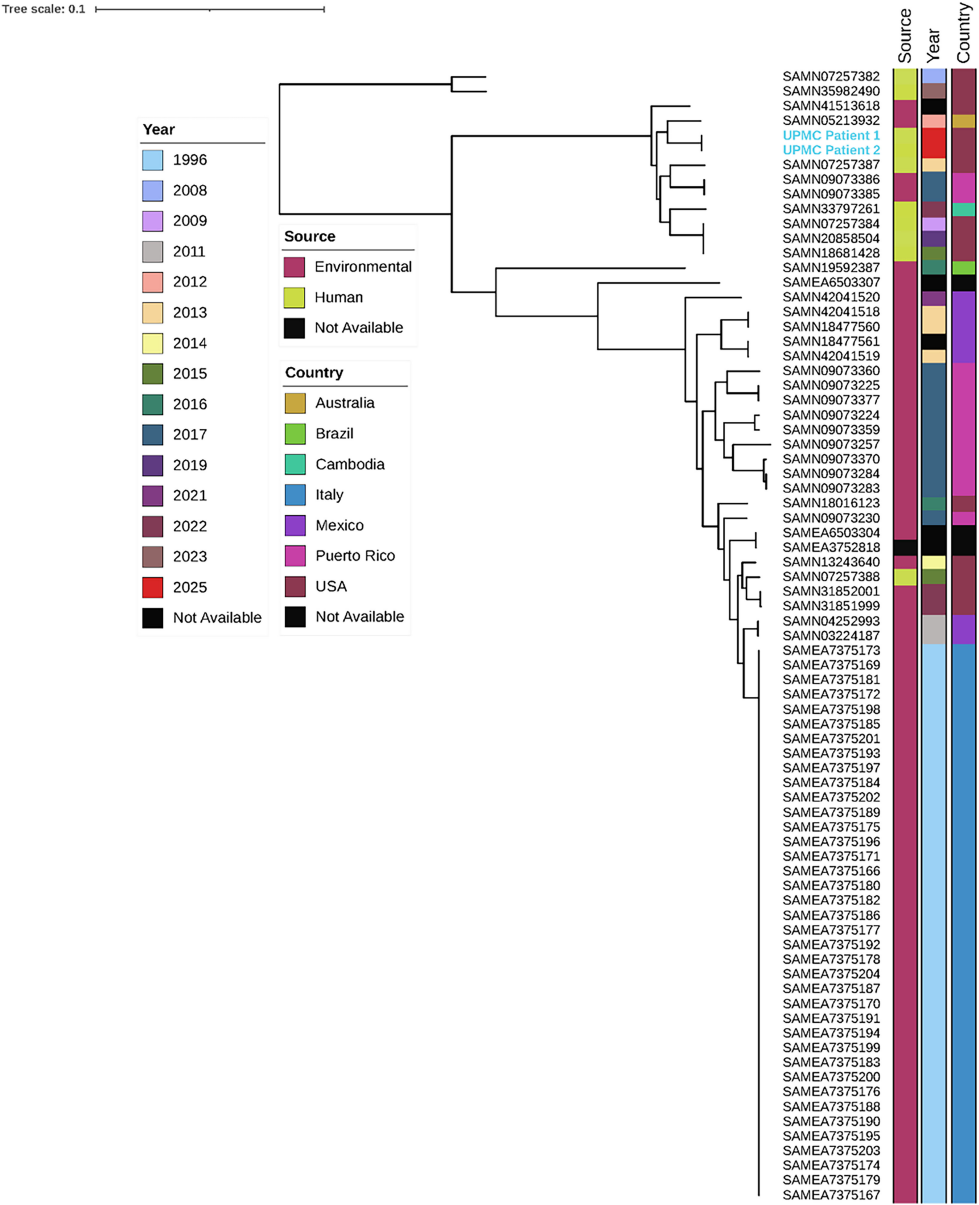
Phylogenetic analysis of UPMC patient *Burkholderia sola* isolates compared to publicly available *B. sola* genomes. UPMC Patient 1 is BC00103, BC00107, and BC00108 and UPMC Patient 2 is BC00106.

The *B. sola* isolates were compared to publicly available Sequence Read Archive *Burkholderia* species genomes, including 76 strains of *B. sola*, and also found to be genetically distinct (>28,000 SNPs to the closest genome). Figure 1 displays a phylogenetic tree comparing patient isolates to the 76 *B. sola* genomes. Of the 76 *B. sola* genomes analyzed, 8 (10.5%) were derived from human sources, suggesting that clinical detection of this species is rare. Most isolates originated from environmental sources across diverse countries, including the United States, Brazil, Mexico, and Italy.^3^

## Discussion

This cluster represents the first reported healthcare-associated transmission of *B. sola*, resulting in clinically significant infection in two lung transplant recipients. Genomic and epidemiologic data support a donor-derived infection in the index patient with subsequent unit-based transmission to a second patient. In the context of *B. cepacia* complex outbreaks, systematic reviews have found that sources of transmission remain unidentified in approximately 25% of cases, and that common reservoirs include respiratory equipment and environmental surfaces—often affecting immunocompromised patients.^4,5^

Routine clinical microbiology initially identified these isolates as *B. cepacia* complex, with speciation as *B. sola* made possible through whole-genome sequencing. Traditional infection prevention efforts often rely on clinical recognition or laboratory detection of unusual organisms.^6^ Whole genome sequencing surveillance may enable early identification of transmission events and improve detection and cessation of rare or emerging pathogens, as demonstrated in this case and in prior work using genomic surveillance.^1,7,8^

## Supporting information

Sundermann et al. supplementary material Sundermann et al. supplementary material

## Supplementary material

To view supplementary material for this article, please visit https://doi.org/10.1017/ice.2025.10261.
